# Surgical outcome of delayed presentation of congenital proximal radioulnar synostosis

**DOI:** 10.1051/sicotj/2015035

**Published:** 2015-12-11

**Authors:** Gaurav Garg, Som P. Gupta

**Affiliations:** 1 S.M.S. Medical College and Attached Hospitals 302004 Jaipur India; 2 Mahatma Gandhi Medical College and Hospital 302022 Jaipur India

**Keywords:** Radioulnar, Synostosis, Radial head excision, Osteotomy, Graft

## Abstract

*Background*: Presentation of proximal radioulnar synostosis varies from cosmetic concerns with no functional limitations to significant pronation deformity which hampers activities of daily living. Surgical management must be considered based on the position of the forearm and functional limitations. We describe the surgical technique, results, and complications of excision of the radial head along with the proximal radius up to the distal extent of the synostosis site and securing the osteotomized radial shaft with a tensor fascia lata graft.

*Materials and methods*: Four patients having six affected elbows with delayed presentation of congenital proximal radioulnar synostosis with dislocated radial head managed surgically were included in the study. There were three males and one female with an average age of 20.25 years (ranging from 16 to 25 years). Preoperatively wrists were locked in the mean pronation position of 51.6° (ranging from 30° to 70°). The indications for surgery were limitation in activities of daily living and an obvious cosmetic deformity.

*Results*: All patients were satisfied with the surgery and showed significant improvement in functional status. Mean active supination was 15° (ranging from 5 to 32°) with passive supination was a mean of 24.8° (ranging from 11° to 44°). Similarly, mean active pronation was 58.5° (ranging from 50° to 71°) with further passive correction up to a mean of 64.16° (ranging from 57° to 87°) at last follow up.

*Conclusions*: This procedure is simple, cost effective, and a reasonable option for treatment of proximal radioulnar synostosis with a dislocated radial head in adult patients. The operation does not require any specialized team or implants, and can be performed in a moderately equipped hospital.

## Introduction

Proximal radioulnar synostosis is a rare upper limb malformation, resulting from the failure of prenatal segmentation of the forearm bones manifesting as a restricted rotational motion of the forearm [1]. Proximal one-third connection is common, with distal radioulnar synostosis extremely rare. Although the exact etiology is not known, a genetic basis can be documented in 25% of cases [2]. It is reported bilaterally in 60–80% of patients [3]. However, it occurs more commonly than perceived as presentation may vary from cosmetic concerns with no functional limitations to significant pronation deformity which hampers activities of daily living [4–6].

Surgical management must be considered based on the position of forearm and functional limitations. There is no established treatment to restore the movements at the radioulnar joint, however, rotational osteotomies distal to the site of synostosis, which provide a more functional position of the forearm, have been advocated especially if the condition is bilateral [7]. Mobilization procedures by separation of the synostosis and placement of a fasciocutaneous graft between the proximal radius and the ulna supplemented with proximal radial osteotomy may benefit some patients by bringing the forearm into a more functional position. Earlier reports on the resection of synostosis in order to restore rotational movements are unsatisfactory with high rate of recurrent fusion [6, 8, 9].

We describe the surgical technique, results, and complications of excision of the radial head along with the proximal radius up to the distal extent of the synostosis site and securing the osteotomized radial shaft with a tensor fascia lata graft.

## Material and methods

**Figure 1.**
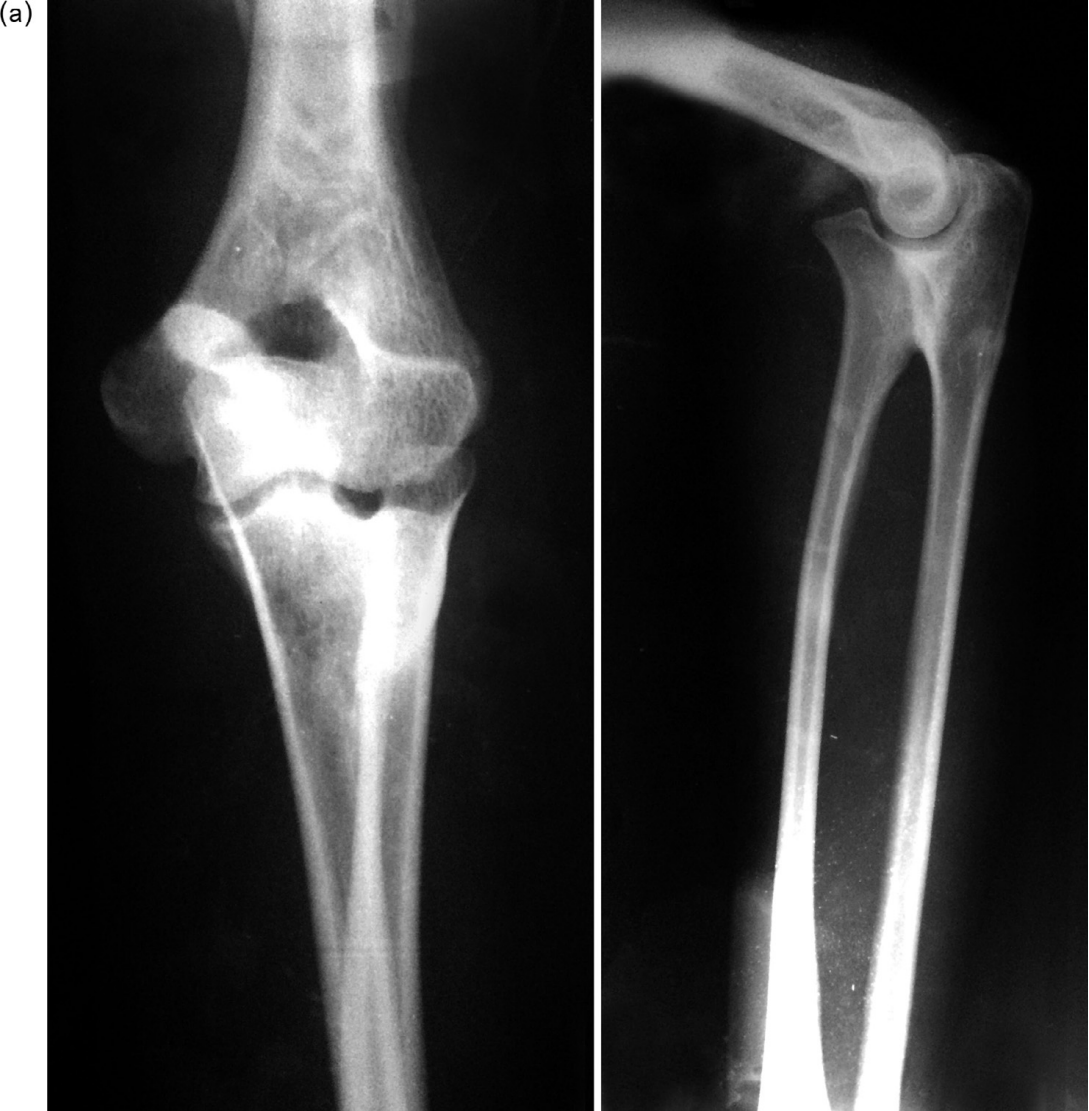
(a) and (b) Bilateral preoperative anteroposterior and lateral radiographs of elbows (Case no. 1), showing proximal radioulnar synostosis with radial head displaced anteriorly consistent with a type-IV deformity [1].

**Figure 2.**
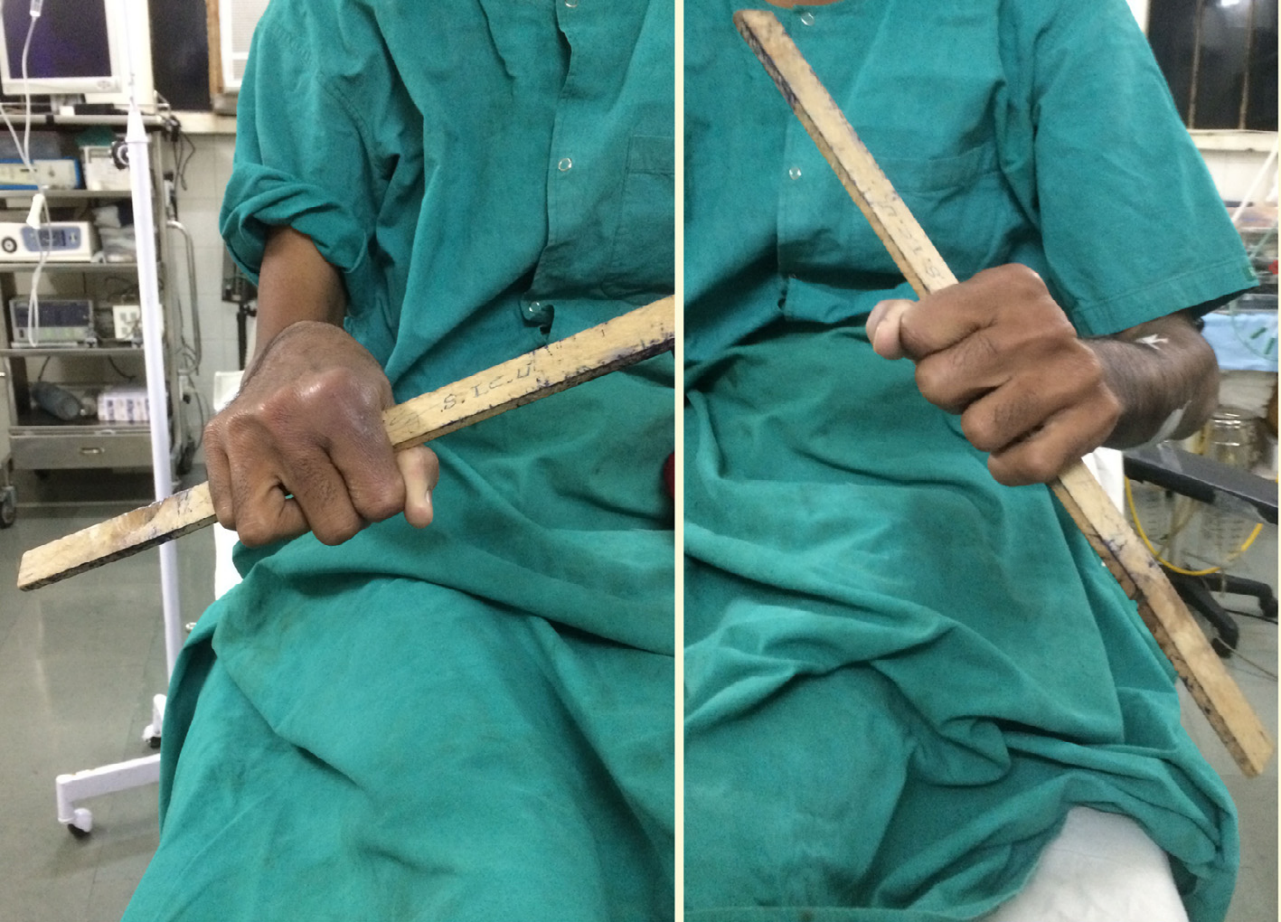
Preoperative photograph showing the elbow fixed in pronation of 70° on right side and 40° on left side.

This was a retrospective study in which medical records of four patients having six affected elbows with delayed presentation of congenital proximal radioulnar synostosis with a dislocated radial head managed surgically between 2009 and 2013 were included. There were three males and one female with an average age of 20.25 years (ranging from 16 to 25 years). Two male patients presented with bilateral deformity, the other two patients had left side involvement although all four patients showed right hand dominance. They were followed up for a mean of 32.1 months (ranging from 16 to 65 months) (Table 1). There were no other concomitant malformations. All patients had deformity since their childhood. The history was elicited by thorough a thorough interrogation of the patients and their guardians. Anteroposterior and lateral radiographs of both forearms with elbow joints were taken (Figures 1a and 1b). Clinical assessment included the ability to perform activities of daily living, degree of rotational deformity of forearm, and the range of motion of elbow and wrist (Figure 2). For measurement of rotational deformity of the forearm, patients were asked to hold a pen or scale in the fist with the elbow held fixed to the side of the chest and at 90° flexion. The angle made between the vertical and the pen/scale was measured with a goniometer.

**Table 1.** Details of patients.

Case no	Age/sex	Side affected	Pronation deformity	Elbow ROM	Clinical type [1]	Total follow-up (months)
1	21/male	Bilateral	R-70°	R-0–130°	R-IV	R-23
			L-40°	L-0–144°	L-IV	L-29
2	26/male	Left	55°	15°–130°	III	38
3	18/male	Bilateral	R-65°	R-0–142°	R- IV	R-16
			L-30°	L-0–140°	L-III	L-22
4	16/female	Left	50°	0–135°	III	65

(R- Right, L- Left).

Preoperatively wrists were locked in the mean pronation position of 51.6° (ranging from 30° to 70°). One patient (Case no. 1) with bilateral involvement had lateral prominence of elbow on the right side with associated elbow pain. One patient (Case no. 2) with pronation deformity of 55° had 15° extension lag of elbow. Two patients (Case nos. 1 and 3) had functional limitations like difficulty in perineal hygiene, inability to hold plates while eating and difficulty in eating with spoon and difficulty in combing hair. Two elbows (Case no. 3 left side, Case no. 4) with pronation deformity of 50° and 30°, respectively, had no functional limitations but they were considered for surgery purely on the basis of cosmetic concern by patients or their guardians. None of the patients had restriction of radio-carpal movements. No neurovascular deficits and no objectively observable muscle atrophy of upper extremity were noted.

The radiographic assessment was based on the classification of Cleary and Omer [1]. Three forearms were classified as type III with visible osseous synostosis associated with posterior dislocation of an hypoplastic radial head. The other three forearms were classified as type IV with anteriorly dislocated radial head. The indications for surgery were limitation in activities of daily living and an obvious cosmetic deformity. Consent was taken from the patients before starting treatment regarding submission of data for publication.

**Figure 3.**
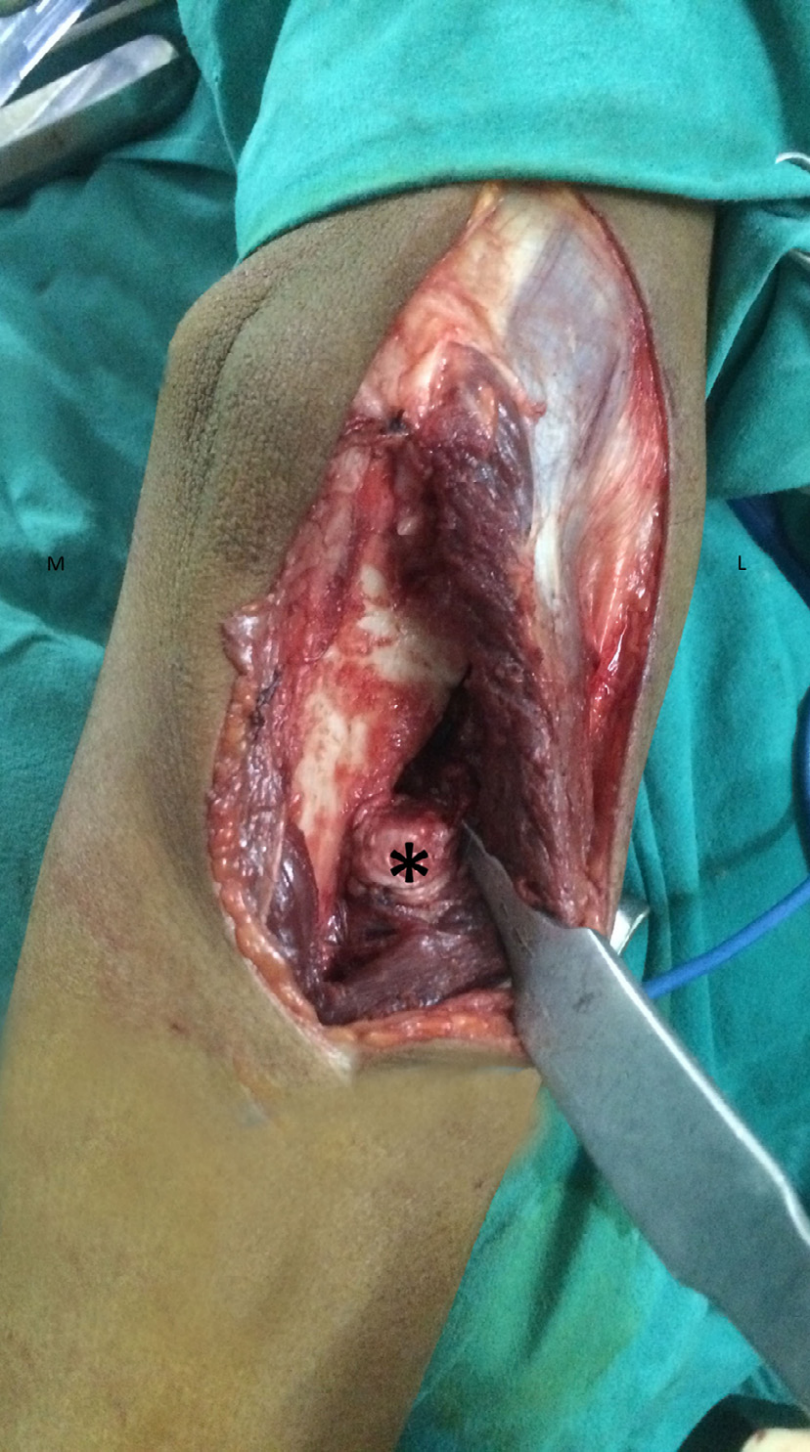
Intraoperative photograph showing excised synostotic site and coverage of osteotomized radial shaft with tensor fascia lata graft (*).

Surgery was performed under general anesthesia in the lateral decubitus position under tourniquet control with the upper arm supported by a padded post with the forearm hanging freely. A posterior curved incision was made, extending 4 cm above the tip of olecranon to the lateral epicondyle and then again curving medially to the lateral border of the ulna. Subperiosteal elevation of muscles along the lateral surface of ulna was done. Once the synostosis site was seen, dissection was carried out proximally. The joint capsule should be opened for proper exposure of the radial head and synostosis site. Using a 2.5 mm drill bit, multiple holes were placed along the synostosis and transversely on the radial shaft distal to the connection. Osteotomy was completed using a sharp osteotome and a large piece of the proximal radius along with the radial head was excised. In two cases (Case no. 1 right side, Case no. 3 right side) even after completing the osteotomy, the radial head was stuck in the surrounding tissue and was removed piecemeal. Around 1 cm of periosteum around the cut end of radius was removed with a chisel. Tensor fascia lata graft measuring 5 cm × 2 cm was taken from the lateral aspect of ipsilateral thigh. The fascial graft was sutured to the cut end of the radius using transverse drill holes (Figure 3). Forearm rotation was checked intraoperatively, ranging from mean pronation of 71.6° to supination of 45.8°. Meticulous closure of the joint capsule should be done to prevent instability. Retracted muscles were sutured to the lateral border of the ulna using two drill holes. The wound was closed in layers.

**Figure 4.**
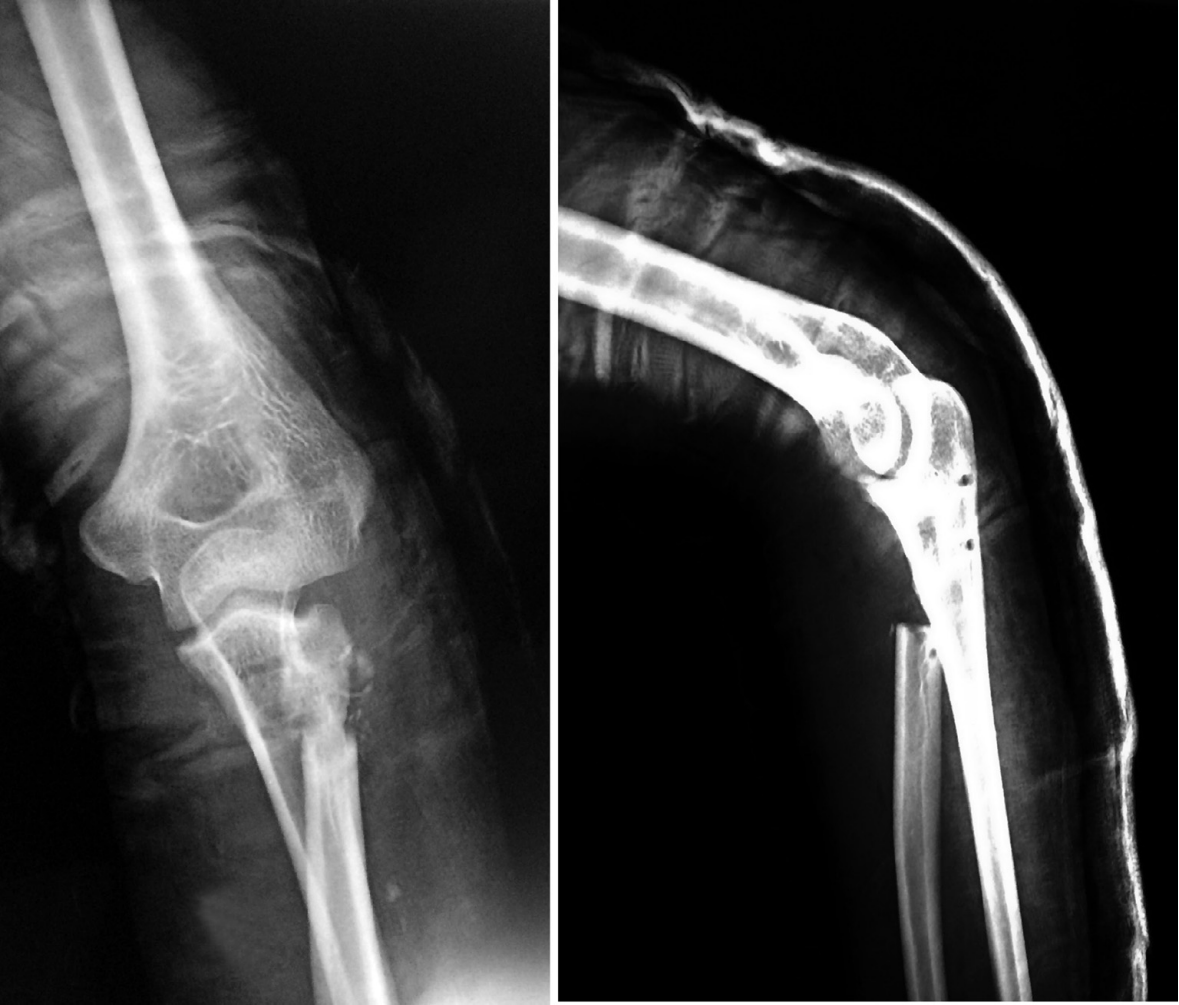
Postoperative radiograph after excision of radial head along with radial shaft up to the distal extent of synostosis. Drill holes seen near the osteotomized radius and ulna for securing the tensor fascia lata graft and muscles, respectively.

**Figure 5.**
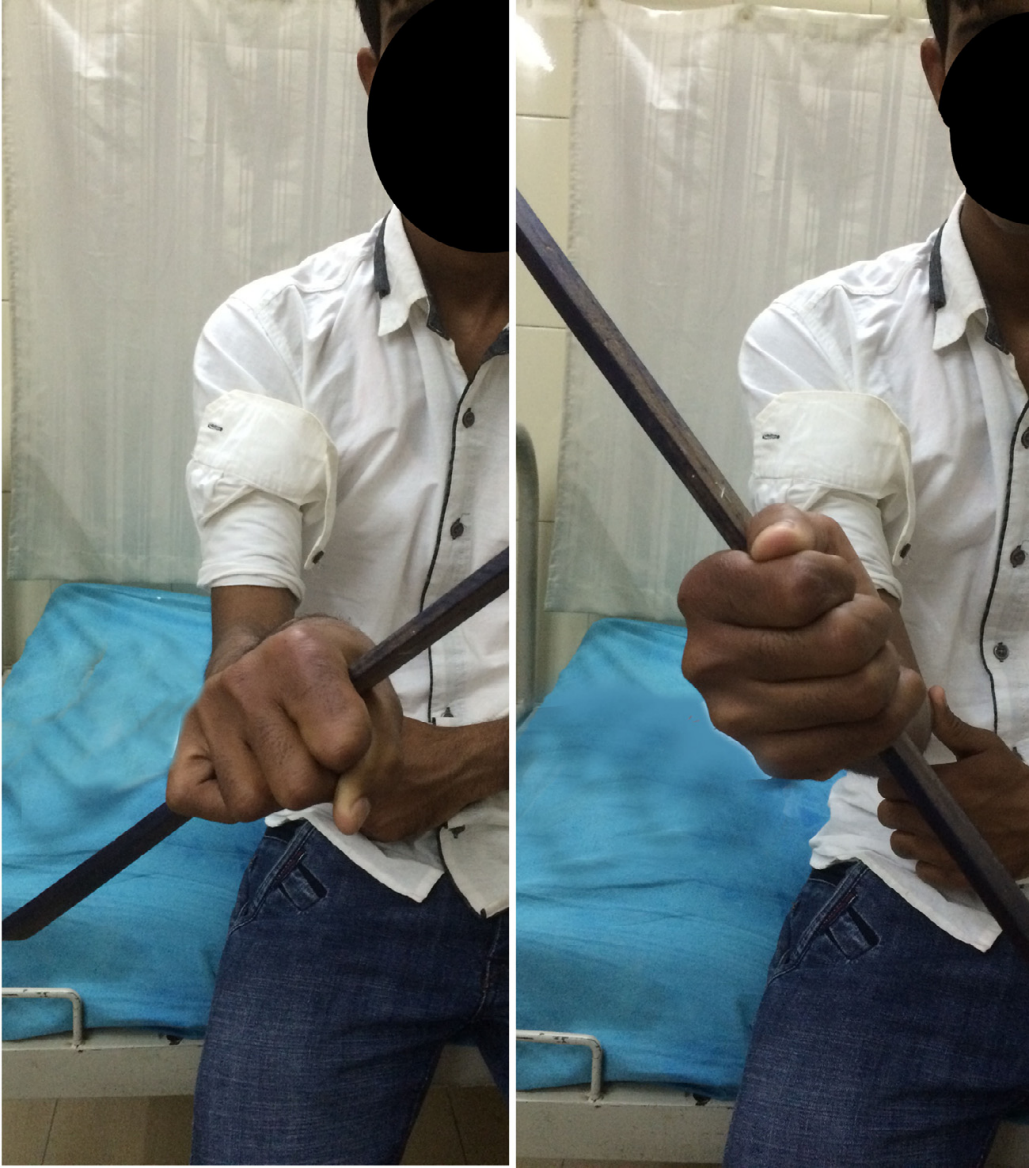
Photographs made almost 2 years after surgery showing good rotational movement of right forearm (Case no. 1).

Postoperatively, the elbow was immobilized in 90° flexion with the forearm in maximum possible supination and held with a posterior above elbow slab. Check X-rays were done postoperatively (Figure 4). The patient was discharged the next day from hospital. Sutures were removed after two weeks and mobilization exercises of elbow and forearm were started. Extensive physiotherapy of the elbow with both active and passive mobilization was started. Patients were then followed one month postoperatively. The range of motion of elbow, wrist, active and passive rotational movements of the forearm was documented. Thereafter, the patients were followed at one month interval for three months, then subsequently at six month intervals. Check X-rays were done at six months and then at yearly interval. At each follow-up, active and passive rotational movements of forearm (Figure 5) along with wrist joint motion were evaluated and patients were subjectively assessed for functional outcome based on the following parameters – perineal hygiene, inability to hold plates while eating and difficulty in eating with spoon and difficulty in combing hair.

## Results

The mean active pronation at one-month follow-up was 58.33° (ranging from 49° to 77°) with further passive correction of a mean of 61.83°(ranging from 52° to 80°). Mean active supination documented at one month was 16° (ranging from 0 to 20°) with a mean passive supination of 32.1° (ranging from 20° to 49°). At the last follow-up, mean active pronation was 58.5° (ranging from 50° to 71°) with further mean passive correction of 64.16° (ranging from 57° to 87°). Similarly, mean active supination was 15° (ranging from 5 to 32°) with a mean passive supination of 24.8° (ranging from 11° to 44°) (Table 2). Mean active flexion of elbow at last follow-up was 129.5° (ranging from 110° to 140°). Two elbows (Case no. 2, Case no. 3 right side) had limitation of elbow extension postoperatively, which resolved subsequently with physiotherapy. There was no change in the range of motion of the wrist during the course of treatment in all patients. Authors were concerned about the wrist pain that patients experience following excision of the radial head in fracture surgery, but fortunately none of the patients reported pain in the wrist joint.

**Table 2.** Serial follow-up of active and passive rotational movements of forearm.

Case no.	Movements at last follow up			
	Active		Passive	
	Pronation	Supination	Pronation	Supination
1	R-61°	R-5°	R-67°	R-11°
	L-54°	L-9°	L-57°	L-20°
2	61°	14°	61°	14°
3	R-54°	R-13°	R-63°	R-22°
	L-50°	L-17°	L-50°	L-38°
4	71°	32°	87°	44°

(R- Right, L- Left).

Two patients with bilateral involvement were first operated for the more severe deformity (right side in both patients) and later underwent second elbow surgery after six months. All patients were satisfied with the surgery and showed significant improvement in functional status. Also, the scar was cosmetically acceptable. One patient (Case no. 1 right side) developed a superficial infection at the suture line, which was dealt with using parenteral antibiotics. The wound subsequently healed. Another patient (Case no. 2) developed a posterior interosseous nerve palsy postoperatively, which recovered completely in eight weeks. One elbow (Case no. 3 right side) with an extension lag preoperatively, had a persistent lag, one month following surgery, but later resolved at the last follow-up.

## Discussion

Proximal radioulnar synostosis is the most common congenital anomaly that functionally impairs the elbow. Failure of longitudinal separation occurs due to persistence of the interzonal mesenchyme between the cartilage anlage of the developing radius and ulna during the seventh week of gestation. The resultant bridge undergoes chondrification, ossification, and eventually may present as a fibrous or bony connection [10]. Genetic influence has been documented through association with positive family history and disorders like Apert’s and Klinefelter syndrome [5], and duplication anomalies of chromosome 14 [7]. It may arise as one of the components of malformation syndrome in children with chromosomal aberrations [11].

Various classifications were proposed based on radiographic features. Wilkie [12] grouped them as type I when there is true bony fusion between radius and ulna proximally and type II when fusion is associated with congenital dislocation of radial head. Cleary and Omer [1] described four radiological types which are more widely accepted, with type III being most commonly seen [13]. These classification systems are more of theoretical significance and their role in planning out treatment has not been proved to a great extent [1, 8].

Treatment is usually tailored to individual needs as most of the patients adapt through increased mobility in wrist and shoulders and so, the indications for surgery are not clearly demarcated. Functional limitations, forearm position, and the aesthetic needs of synostosis must be given weight when considering surgery. Ogino and Hikino [14] concluded in their study, that the mean fixed pronation of patients with disability was 60° and for patients without complaints it was 20°. Patients with less than 30° of fixed pronation generally do not require surgery. For those between 30° and 60° of fixed pronation, one must carefully individualize treatment. Surgery must be considered for patients with more than 60° of fixed pronation deformity [1].

Management of proximal radioulnar synostosis is technically difficult, physically and psychologically demanding for the patient with unpredictable results. Various surgical techniques like resection of the anastomosis [12], insertion of a swivel apparatus, reconstruction with interpositional materials [5], and rotational osteotomy through or distal to the synostosis mass [15] have been proposed. Results of resection of the synostosis were not convincing. Rotational osteotomies to position the forearm in a more functional position are well accepted with good outcomes. The reported complications include loss of correction, vascular complications due to undue stress during correction, and delayed union [5, 13–15]. The early results using free vascularized fascial fat grafts after mobilization of the synostosis with corrective osteotomy of the radius were more promising but they require microvascular anastomosis which is difficult to achieve in a moderately equipped hospital [16].

Our technique of excision of radial head along with the proximal radius up to the distal extent of the synostosis site and securing the osteotomized radial shaft with a tensor fascia lata graft is technically easy to perform and leaves a cosmetically acceptable posterior scar compared to the lateral approach. An implant was not used so there was no concern about non-union. The operation can be performed in an averagely equipped hospital setup. Our study was limited as we have not objectively compared the pre- and postoperative functions, like using the hand function test of Bhatt and Mehta [17] and the authors have a general consensus that it requires a long-term follow-up to provide firm evidence.

This study helps us to conclude, that excision of the radial head along with the proximal radius up to the distal extent of the synostosis site and securing the osteotomized radial shaft with a tensor fascia lata graft is simple, cost effective, and is a reasonable option for the treatment of proximal radioulnar synostosis with a dislocated radial head in adult patients. The operation does not require any specialized team and implants, and can be performed in a moderately equipped hospital.

## Conflict of interest

Authors report no conflict of interest, financial or otherwise, concerning the material or methods used in this study or the findings specified in this paper. There were no sources of financial or material support for this report.

## References

[1] Cleary JE, Omer GE (1985) Congenital proximal radio-ulnar synostosis: natural history and functional assessment. J Bone Joint Surg Am 67-A, 539–545. 3980498

[2] Yamnime K, Salon A, Pouliquen JC (1998) Congenital radioulnar synostosis. Study of a series of 37 children and adolescents. Chir Main 17(4), 300–308. 10855298

[3] Lescault E, Mulligan J, Williams G (2000) Congenital radioulnar synostosis in an active duty soldier: case report and literature review. Mil Med 165(5), 425–428. 10826394

[4] Dogra BB, Singh M, Malik A (2003) Congenital proximal radioulnar synostosis. Indian J Plast Surg 36, 36–38.

[5] Simmons BP, Southmayd WW, Riseborough EJ (1983) Congenital radioulnar synostosis. J Hand Surg 8, 829–838. 10.1016/s0363-5023(83)80078-1 6643957

[6] Hausen OH, Andersen NO (1970) Congenital radioulnar synostosis. Report of 37 cases. Acta Orthop Scand 41, 225–230. 5486179 10.3109/17453677008991509

[7] Ramchandran M, Lau K, Jone DHA (2005) Rotational osteotomies for congenital radioulnar synostosis. J Bone Joint Surg Br 87-B, 1406–1410. 10.1302/0301-620X.87B10.16445 16189317

[8] Miura T, Nakamura R, Suzuki M, Kanie J (1984) Congenital radio-ulnar synostosis. J Hand Surg 9-B, 153–155. 6747418

[9] Sachar K, Akelman E, Ehrlich MG (1994) Radioulnar synostosis. Hand Clin 10, 399–404. 7962146

[10] Mital MA (1976) Congenital radioulnar synostosis and congenital dislocation of the radial head. Orthop Clin North Am 7, 375–383. 1264432

[11] Cho YG, Kim DS, Lee HS et al. (2004) A case of 49,XXXXX in which the extra X chromosomes were maternal in origin. J Clin Pathol 57(9), 1004–1006. 15333671 10.1136/jcp.2004.017475 PMC1770429

[12] Wilkie DPD (1914) Congenital radioulnar synostosis. Br J Surg 1, 366–375.

[13] Murase T, Tada K, Yoshida T, Moritoma H (2003) Derotational osteotomy at the shafts of the radius and ulna for congenital radioulnar synostosis. J Hand Surg Am 28, 133–137. 12563650 10.1053/jhsu.2003.50010

[14] Ogino T, Hikino K (1987) Congenital radio-ulnar synostosis: compensatory rotation around the wrist and rotation osteotomy. J Hand Surg Br 12, 173–178. 3624970 10.1016/0266-7681_87_90006-4

[15] Green WT, Mital MA (1979) Congenital radio-ulnar synostosis: surgical treatment. J Bone Joint Surg 61A, 738–743. 457717

[16] Jebsen RH, Taylor N, Trieschmann RB, Trotten MJ, Howard LA (1969) An objective and standardized test of hand function. Arch Phys Med Rehabil 50, 311–319. 5788487

[17] Bhatt CR, Mehta CD (2011) Case report: congenital radioulnar synostosis and its embryological correlation and functional assessment. J Anat Soc India 60(2), 236–238.

